# *OsDOF11* Affects Nitrogen Metabolism by Sucrose Transport Signaling in Rice (*Oryza sativa* L.)

**DOI:** 10.3389/fpls.2021.703034

**Published:** 2021-09-10

**Authors:** Xinglei Huang, Yiyan Zhang, Leilei Wang, Xinyi Dong, Weixin Hu, Min Jiang, Gang Chen, Gynheung An, Fei Xiong, Yunfei Wu

**Affiliations:** ^1^Jiangsu Key Laboratory of Crop Genetics and Physiology/Co-Innovation Center for Modern Production Technology of Grain Crops/Joint International Research Laboratory of Agriculture and Agri-Product Safety, Yangzhou University, Yangzhou, China; ^2^Jiangsu Key Laboratory of Crop Genomics and Molecular Breeding/Jiangsu Co-Innovation Center for Modern Production Technology of Grain Crops, Yangzhou University, Yangzhou, China; ^3^Crop Biotech Institute and Graduate School of Biotechnology, Kyung Hee University, Yongin, South Korea

**Keywords:** *OsDOF11*, nitrogen, water content, stress, rice

## Abstract

Carbon and nitrogen antagonistically regulate multiple developmental processes. However, the molecular mechanism affecting nitrogen metabolism by sucrose transport remains poorly defined. Previously, we noted that *Oryza sativa DNA BINDING WITH ONE FINGER 11* (*OsDOF11*) mediated sucrose transport by binding to the promoter regions of *Sucrose Transporter 1* (*SUT1*), *Oryza sativa Sugars Will Eventually be Exported Transporters 11 (OsSWEET11)*, and *OsSWEET14*. Here, we note that *OsDOF11* promotes nitrogen uptake and then maintains the ratio of fresh weight to dry weight in seedling plants and the effective leaf blade at flowering stages. Mutants of the sucrose transporter gene *OsSWEET14* displayed a phenotype similar to that of *OsDOF11*. By microarray analysis and qRT-PCR in *OsDOF11* mutant plants, *OsDOF11* affected the transcription level of amino acid metabolism-related genes. We further found that mainly amino acid contents were reduced in flag leaves but increased in seeds. Both sugar and organic nitrogen changes caused the ratio of fresh weight to dry weight to decrease in *OsDOF11* mutant seedling plants and mature leaves, which might result in vigorous reduced metabolic activity and become less susceptible to stress. These results demonstrated that *OsDOF11* affected nitrogen metabolism by sugar distribution in rice, which provided new insight that *OsDOF11* coordinated with C and N balance to maintain plant growth activity.

## Introduction

Rice (*Oryza sativa* L.) is one of the most important crops for over one-third of the population of the world (Sasaki and Burr, 2000). With the rapid development of society, the population has increased faster year by year, and the global human population was estimated to be near 7.7 billion in 2021, which caused a world food crisis. To some degree, an increase in photosynthetic rate or a higher nitrogen use efficiency in plants and crops might be a vital element to resolve the food crisis. Sucrose, the primary product of photosynthesis, plays a pivotal role in mediating various physiological processes and mainly provides raw materials that participate in starch synthesis (Nakamura, 2002; Fitzgerald et al., 2009; Tian et al., 2009; Yan et al., 2011; Feng et al., 2019). Sucrose is synthesized in mesophyll cells and then moves from source tissue into sink tissue cells via a phloem loading system to support plant growth and development (Ayre, 2011). Sucrose phloem loading systems contain two traditional sucrose transport routes, apoplastic loading and symplastic loading (Braun et al., 2014). These transport pathways always initiate from mesophyll cells by bundle sheath, mestome sheath, and PP (phloem parenchyma) and enter PP-TSEs (thick-walled sieve elements) (symplastic loading) or CC-SEs (companion cells-sieve elements) (apoplastic loading) in grasses (Eom et al., 2012; Braun et al., 2014). Symplastic loading always depends on plasmodesmata. However, two membrane protein family genes, *SUT* (*Sucrose Transporter*, also called *SUC* for *Sucrose Carrier*) and *SWEETs* (***Sugars Will Eventually be Exported Transporters)-type transporter***, participate in apoplastic loading between CC and SE, except for plasmodesmata (Braun et al., 2014; Wu et al., 2018).

As sucrose transporters mediate long-distance sucrose phloem loading, several transcription factors regulate its expression levels. Bai et al. (2016) isolated a gene (Os02g0725900) encoding nuclear factor *Y B1* (*NF-YB1*) expressed in caryopses from 4 to 21 DAP, as previously reported (Sun et al., 2014; Bai et al., 2016). Mutations of *OsNF-YB1* by CRISPR/cas9 and RNA interference (RNAi) transgenic plants showed a chalky phenotype due to binding to the promoters of the sucrose transporter genes *Oryza sativa Sucrose Transporter 1* (*OsSUT1*), *OsSUT3*, and *OsSUT4* (Bai et al., 2016). *Oryza sativa DNA BINDING WITH ONE FINGER 11* (*OsDOF11*), a transcription factor, is a sucrose transport inducer. Mutations of *OsDOF11* and RNAi transgenic plants displayed semi-dwarf, fewer tillers, smaller panicles and smaller grain sizes due to lower levels of sucrose transport activity. The expression of four *SUT* genes – *OsSUT1*, *OsSUT3*, *OsSUT4*, and *OsSUT5* – as well as two SWEET genes, *OsSWEET11* and *OsSWEET14*, was reduced in various organs of the mutant, including the germination seeds, seedlings, leaves, stems, and young panicles. *OsDOF11* binds directly to the promoter regions of *SUT1*, *OsSWEET11*, and *OsSWEET14* (Wu et al., 2018). However, the *OsSUT1 Tos17* insertional mutant does not produce homozygous seeds, *OsSWEET11* involves stamen development, and the *OsSWEET14* mutant displays a similar phenotype with a dwarf phenotype and smaller seeds (Yang et al., 2018). In addition, *DOF11-VP16-Myc* driven by the 2.0 kb *DOF11* promoter rescued the *DOF11* semi-dwarf phenotype (Kim et al., 2021).

Mineral elements are required for plant growth and development, and most of these mineral elements are needed to synthesize the necessary organic substances for plant life. Plants possesses an intricate regulatory mechanism that coordinates the capacity of carbon (C) assimilation with nitrogen (N) metabolism (Nunes-Nesi et al., 2010). C, which is mainly assimilated by fixed CO_2_ and water under photosynthesized processes into catalytically synthesized carbohydrates and then utilized to build the main skeleton of proteins, nucleotides of DNA or RNA, lipids, numerous metabolites and cellular components, is one of the most critical elements in plants. N is taken by the roots and then assimilated in the roots and shoots. Phosphoenolpyruvate carboxylase (PEPC) plays a vital role in the carboxylation of phosphoenolpyruvate to form oxaloacetate (Wu et al., 2017). Glutamine synthetase is coupled with glutamate synthase (GOGAT) in the GS/GOGAT cycle. *OsNADH-GOGAT1* is expressed at root tips, leaves and seeds, while *OsNADH-GOGAT2* is predominantly expressed in mature leaves (Tamura et al., 2011). Nitrogen assimilation is integrated with carbohydrate distribution. Accordingly, carbohydrate allocation between the synthesis of organic acids and starch and sucrose is noticeably affected by nitrogen metabolism at the transcript and post-translation levels (Coruzzi and Zhou, 2001). Recently, great achievements have been made in the dissection of C and N metabolism to control plant developmental processes (Wang et al., 2020; Zhang et al., 2020). However, little is known about whether sucrose transport activity is involved in amino acid metabolism. Here, we report that mutation in *OsDOF11* causes the ratio of fresh weight to dry weight to decrease due to abnormal nitrogen uptake and affects nitrogen metabolism.

## Materials and Methods

### Plant Materials and Growth Conditions

Previously, we reported that T-DNA insertion Within the first intron of *OsDOF11* in a background of cultivar Dongjin (*Oryza sativa* L. ssp. *japonica*) caused a null allele (Jeon et al., 2000). and we selected *RNAi-9* From 12 independently *OsDOF11-RNAi* plants With same background of *OsDOF11* T-DNA insertion line (Wu et al., 2018). the *OsSWEET14* mutant line (Line PFG_3D-03008), T-DNA insertion Within the first intron, Was derived From cultivar Hwayoung (*Oryza sativa* L. ssp. *japonica*) (Jeong et al., 2006; Antony et al., 2010). Seeds Were germinated at 28°C for 7 days Under Yoshida medium (with or without 3% sucrose) With multiple concentrations of nitrogen (normal N, 0 N, and 10% N) (Yoshida et al., 1976; Wu et al., 2017). the seedlings Were cultivated in pools With 3 different concentrations of nitrogen (lower concentration: treatment With 90 kg/hm^2^ urea; middle concentration: treatment With 135 kg/hm^2^ urea; higher concentration: treatment With 180 kg/hm^2^ urea) at the seedling stage, tillering stage, and flowering stage in Yangzhou University, China. the whole plant at the seedling stage in Yoshida medium, flag leaves at the flowering stage and seeds in pools With 3 different concentrations of nitrogen of WT and *osdof11-1* plants, as well as RNAi-9, Were harvested.

### Transcriptome Analysis

Three individual sample were used for transcriptomic analysis. Total RNA was extracted from the main tiller flag leaves of WT and *osdof11-1* plants. The samples were separated, frozen immediately in liquid nitrogen, and stored at −80°C before RNA isolation. Total RNA isolation was conducted using an Ultrapure RNA Kit (CWBIO, CW0581S). The mRNA of each sample was enriched using oligo(dT) magnetic beads and digested into short fragments by fragmentation buffer. Random hexamers were used as primers for first-strand and second-strand cDNA synthesis. These double-stranded samples were treated with T4 DNA polymerase and T4 polynucleotide kinase for end-repairing and dA-tailing, followed by T4 DNA ligase treatment for adaptor ligation. Afterward, fragments approximately 200 bp long were collected and used as templates for PCR amplification to create the cDNA library. This library was pair-end sequenced using the PE90 strategy (paired-end reads of 90 base pairs per read) on an Illumina HiSeq^TM^ 2,000. The raw reads were processed to generate clean-read datasets by removing the adaptor sequences, reads with > 5% ambiguous bases (noted as N), and low-quality reads that contained more than 20% of bases with qualities of < 20. The clean reads were then aligned to the rice japonica genome (version: Tigr 7.0) using the Tophat program (v2.0.11) under the following parameters: -a 10 -m 0 -i 31 -I 500000 –G. The DEseq algorithm was applied to filter the differentially expressed genes (Yang et al., 2018). In this study, genes with a *q*-value < 0.05 and a fold-change > 2 between the control and treated samples were considered significantly differentially expressed.

### GO Analysis

The GO information of differentially expressed genes was retrieved from the rice oligonucleotide array database^1^ (Cao et al., 2012). Fold-enrichment values were calculated by dividing the query number by the query expected value. We selected GO terms with a fold enrichment greater than 2 and a hypergeometric *P*-value below 0.05. Visualization of GO terms was performed by Microsoft excel, and Illustrator software was used to polish GO terms. Functional information for differentially expressed genes was analyzed by the MapMan toolkit (3.6.0RC1), which has been used frequently for functional classification of transcriptome data (Thimm et al., 2004).

### RT–PCR Analyses

Total RNA was isolated from flag leaf blades of greenhouse-grown plants at the flowering stage. The cDNAs were synthesized and quantitative real-time qRT-PCR was performed as previously described. The internal control was rice *OsUBQ5* (*LOC_Os01g22490*). All experiments were conducted at least three times, with three or more samples taken at each point. To ensure primer specificity, we performed the experiments when the melting curve showed a single sharp peak. The PCR products were sequenced to verify the specificity of the reaction (Wu et al., 2018). SYBR Green (Invitrogen) were 50°C for 2 min, 95°C for 2 min, and 40 cycles of 95°C for 15 s and 57°C for 30 s. The amount of product was quantified using a standard curve after normalization with transcripts from an actin gene (*OsUBQ5*). All primers used for studying gene expression are listed in Supplementary Table 2.

### Nitrogen Content

The seedling plant and effective leaf blade were harvested, ground in liquid nitrogen, and then filtered through a 100-μm sieve. The total nitrogen content was measured using a CHN-Nitrogen analyzer (Vario EL cube, Elementar Analysensysteme Gmbh) and converted to protein content by using a conversion factor of 6.25 (Mariotti et al., 2008). The protein contents in the seedling plant and effective leaf blade were calculated on a dry basis.

### Extraction, Purification, and Quantification of Amino Acids

The flag leaf blades at the heading stage and the mature seeds from transgenic plants, as well as WT, were harvested from the main tiller of each plant. For total amino acid analysis, 10mg of rice power of each sample was hydrolyzed with 1mL of 6N HCl (Sigma, United States) in a 2mL screw-cap tube before adding 10nmol L-(+)-norleucine (Wako Pure Chemicals, Japan). The samples were then heated at 110°C for 24h, followed by the treatment of 6h at 65°C in order to evaporate HCl completely. The residue was then dissolved in 1mL Na-S^TM^ buffer and centrifuged at 1,600 × *g* for 10min at room temperature. The supernatant was filtered with a 0.45μm nylon membrane syringe filter (Pall Life Sciences, United States) and transferred to an autosampler bottle for amino acid analysis. HPLC data were normalized with the level of L-(+)-norleucine per sample. Three biological replicates were designed for each sample. Seventeen amino acids were measured, including alanine (Ala), arginine (Arg), aspartic acid (Asp), cysteine (Cys), glutamic acid+glutamine (Glu), glycine (Gly), histidine (His), isoleucine (Ile), leucine (Leu), lysine (Lys), methionine (Met), phenylalanine (Phe), proline (Pro), serine (Ser), threonine (Thr), tyrosine (Tyr), and valine (Val) (Yang et al., 2018).

### Content of Amylose Determination and Soluble Sugar

Starch was defatted using 85% methanol and then dissolved in dimethyl sulfoxide containing urea solution. The iodine absorption spectrum was scanned from 400 to 900 nm using a spectrophotometer (Ultrospec 6300pro, Amersham Biosciences, United Kingdom). The AAC (apparent amylose content) was calculated from the absorbance at 620 nm by reference to a standard curve. The true AC (amylose content) was measured using a Megazyme Amylose/Amylopectin Assay Kit (K-AMYL) according to the manufacturer’s instructions. The amylose/amylopectin ratio was calculated based on AC. The soluble sugar content was determined according to Stitt et al. (1989). The amylose and soluble sugar content in the seedling plant and effective leaf blade was calculated on the basis of dry weight.

### Statistical Analysis

Student’s *t*-test by Excel 2010 (Microsoft, United States) was performed to determine any statistically significant differences among values measured from WT, *osdof11-1*, and *RNAi-9* samples in each experiment. Each data point represents the mean from at least four different plants. *T*-test was used to compare means at a significance level of *P* < 0.05 or *P* < 0.01.

## Results

### *OsDOF11* Promotes Nitrogen Assimilation by Sucrose Transport

**FIGURE 1:**
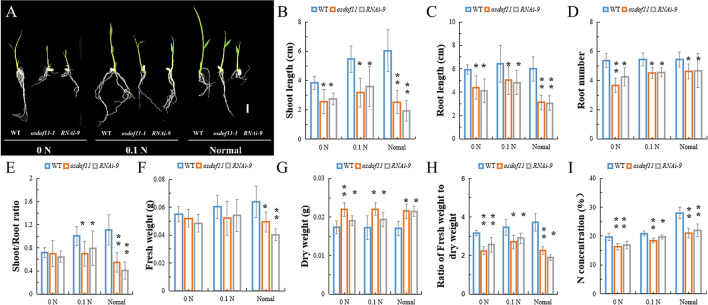
Characterization of the *OsDOF11* mutants at the seedling stage under multiple N contents in Yoshida medium. **(A–H)** Phenotypes of the *odof11-1* mutant, *OsDOF11-RNAi* plants, and WT under multiple N concentrations of Yoshida medium. **(A)** Picture of seedling plants at 7 days after germination; **(B)** shoot length; **(C)** root length; **(D)** root number; **(E)** ratio of shoot to root length; **(F)** fresh weight; **(G)** dry weight, **(H)** ratio of fresh weight to dry weight; **(I)** N concentration. 0 N: Yoshida medium without nitrogen, 0.1 N: Yoshida medium with 10% nitrogen, and normal medium: Yoshida medium with a normal content of nitrogen. Scale bar = 1 cm. Error bars represent SE of at least five samples. *P < 0.05; **P < 0.01.

**FIGURE 2:**
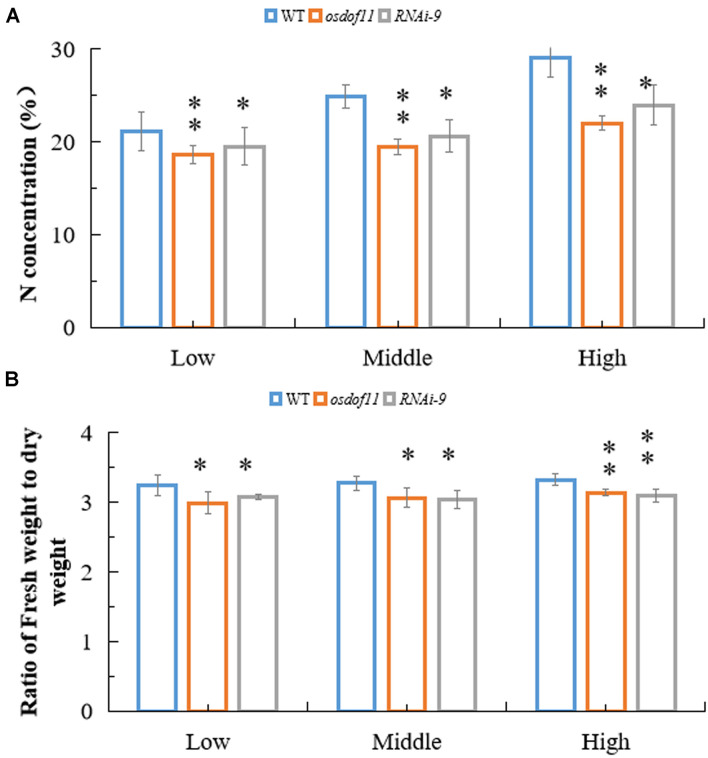
*OsDOF11* affects nitrogen metabolism by sugar distribution in the effective leaf blade at the flowering stage. **(A)** Ratio of fresh weight to dry weight of effective leaf blade at flowering stage; **(B**) N concentration in effective leaf blade at flowering stage; Low: treatment by urea 90 kg/hm^2^; Mid: treatment by urea 135 kg/hm^2^; High: treatment by urea 180 kg/hm^2^. Error bars represent SE of at least five samples. *P < 0.05; **P < 0.01.

Sucrose not only acts as a type of carbohydration for providing energy, but is also utilized to synthesize other organic materials. We described that *OsDOF11* participated in sugar distribution (Wu et al., 2018). To further analyze the function of *OsDOF11*, we chose T-DNA insertion line *osdof11-1* and *OsDOF11* RNA interference line 9 (*RNAi-9*), in which *OsDOF11* transcription level were reduced (Wu et al., 2018). Mutants of *OsDOF11* and background line of cultivar Dongjin (WT) were grown in Yoshida medium without sucrose but with multiple concentrations of nitrogen, which contains ammonium and nitrate (0 N, 10% N and normal N). The images of the cultivated rice at 7 DAG are shown in Figure 1A. As demonstrated, the shoot length, root length, crown root number and fresh weight were reduced in the *osdof11-1* line, as well as in *RNAi-9* (Figures 1B–F). However, the dry weight was increased, which caused a reduction in the ratio of the fresh weight to dry weight in mutants of *OsDOF11* (Figures 1G,H). As the nitrogen content increased in the medium from 0 N to normal N, more nitrogen was taken by WT seedling plants (Figure 1I). Compared with WT, nitrogen concentrations were reduced in both the *OsDOF11* and *RNAi-9* lines. To confirm this result, we grew *OsDOF11*-related material in paddy fields with different concentrations of N by urea at the seedling, tillering, and filling stages (lower concentration: 90 kg/hm^2^; middle concentration: 135 kg/hm^2^; higher middle concentration: 180 kg/hm^2^). We measured the N concentration per gram of dry weight (DW) directly in mutants of *OsDOF11* leaf blade and WT rice at the flowering stage. We found that the effective leaf blades of *OsDOF11* mutants had lower N content under different nitrogen treatments (low N, middle N, and high N) (Figure 2A). Additionally, the ratio of fresh weight to dry weight was also reduced in all the mutants (Figure 2B). These results are similar to the results at the seedling stage.

### OsDOF11 Promotes Nitrogen Assimilation by *OsSWEET14*

**FIGURE 3:**
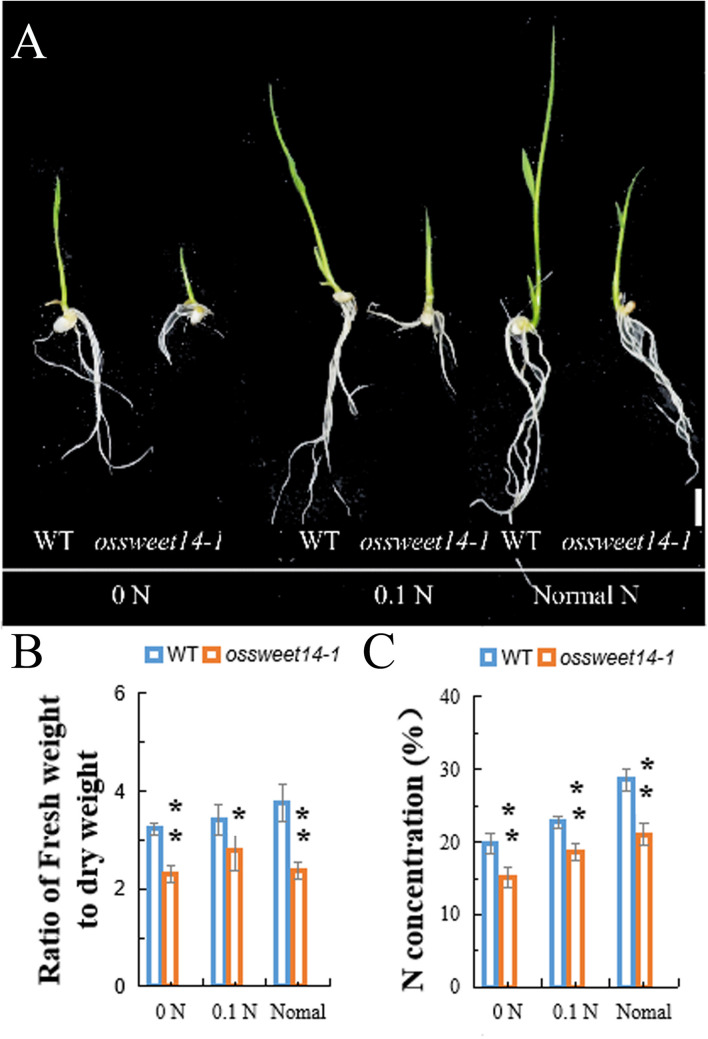
Characterization of the *OsSWEET14–1* mutant at the seedling stage under multiple N contents of Yoshida medium without sucrose. **(A–C)** Phenotypes of *OsSWEET14–1* and WT under multiple N concentrations in Yoshida medium. **(A)** Picture of seedling plants at 7 days after germination; **(B)** ratio of fresh weight to dry weight; **(C)** N concentration. 0 N: Yoshida medium without nitrogen, 0.1 N: Yoshida medium with 10% nitrogen, and normal medium: Yoshida medium with a normal content of nitrogen. Scale bar = 1 cm. Each data point represents the mean (±SE) from at least four different plants. *P < 0.05; **P < 0.01.

Previously, we noted that *OsDOF11* mediated sucrose transport by binding to the promoters of the sucrose transporter genes *OsSUT1*, *OsSWEET11*, and *OsSWEET14* (Wu et al., 2018). We added 3% sucrose to Yoshida medium with multiple concentrations of nitrogen and repeated the growth of the *OsDOF11* mutant experiments. The trend was similar in Yoshida medium with or without sucrose (Supplementary Figure 2). Interestingly, as sucrose was added to Yoshida medium, the WT took slightly more nitrogen than the WT without sucrose. Thus, we analyzed the phenotype of *OsSWEET14* mutant (Supplementary Figure 1). We found that the nitrogen content and ratio of fresh weight to dry weight in the *OsSWEET14* mutant were reduced under multiple concentrations of nitrogen (Figure 3). These results suggest that *OsDOF11* mediates nitrogen uptake or assimilation by sucrose transport.

### Transcriptome Analysis of *OsDOF11* Mutant

**FIGURE 4:**
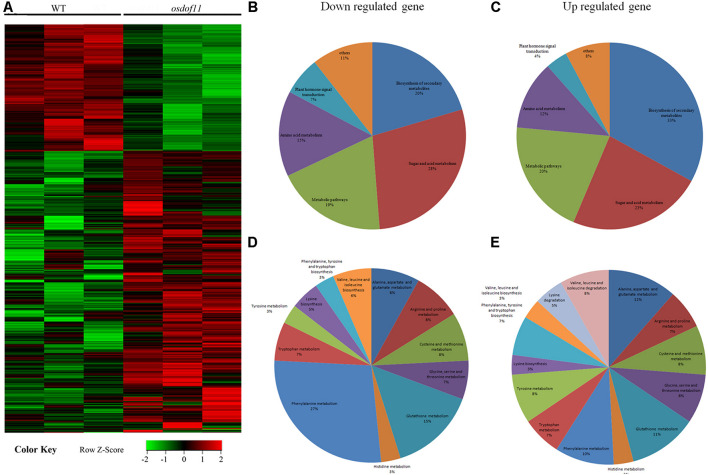
Microarray analysis of related genes in WT and *OsDOF11* mutants. **(A)** Microarray analysis; **(B)** Down regulated genes; **(C)** up regulated genes; **(D)** down regulated genes related to amino acid metabolism; **(E)** Up regulated genes related to amino acid metabolism.

**FIGURE 5:**
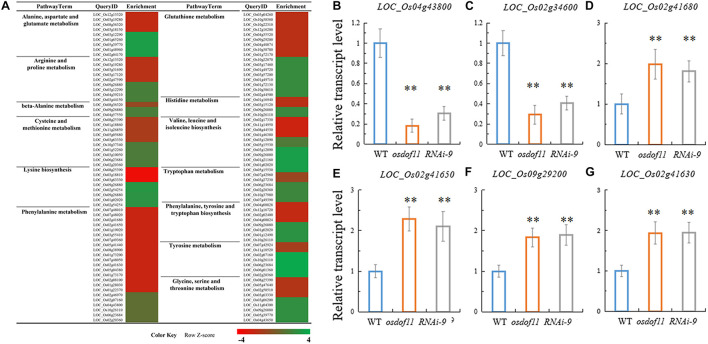
Transcription analysis of N-related genes in WT and *OsDOF11* mutants. **(A)** Microarray analysis of amino acid-related genes in WT and *OsDOF11* mutants. **(B–G)** Expression levels of N assimilation genes relative to *OsUBQ5* evaluated at flowering stages in leaf blades. **(B)** LOC_Os04g43800; **(C)** LOC_Os02g34600; **(D)** LOC_Os02g41680; **(E)** LOC_Os02g41650; **(F)** LOC_Os09g29200; **(G)** LOC_Os02g41630. Each data point represents the mean (±SE) from at least four different plants. **P < 0.01.

Previous studies have reported that decreased sucrose transport activity caused increased sugar content of leaves and reduced grain size (Coruzzi and Zhou, 2001; Eom et al., 2012; Julius et al., 2017). During plant development, the carbon skeleton driven by the photosynthesis product influences the activity of enzymes for N metabolism, especially amino acids. The flag leaf transported and reorganized nutrition, which contributed mainly to grain development (Reguera et al., 2013). We first tried to investigate nitrogen-related genes affected in the *OsDOF11* mutants by transcriptome analysis using mRNAs prepared from the flag leaf blades of *osdof11-1* and WT at the heading stage. The RNA sequencing data identified 22,282 annotated genes, among which 930 had at least twofold higher transcript levels, while 880 had at least twofold lower levels in *osdof11-1* compared with WT. These genes were involved in multiple signaling pathways, especially sugar and acid metabolism, amino acid metabolism, metabolic pathways, plant hormone signal transduction, biosynthesis of secondary metabolites, and others (Figure 4). The top 20 enriched pathways were described in Supplementary Figure 3. Seventeen fundamental amino acid metabolism-related genes were changed (Figure 5A). Among the changed genes, 132 genes were downregulated, and 114 genes were upregulated (Supplementary Table 1). We therefore selected N signaling genes to verify the results of the RNA sequencing experiment. From that group of genes, we randomly selected six N-related genes, of which two were downregulated (LOC_Os04g43800 and LOC_Os02g34600) and four were upregulated (LOC_Os02g41680, LOC_Os02g41650, LOC_Os09g29200, and LOC_Os02g41630). Quantitative real-time RT-PCR analyses confirmed the microarray results (Figures 5B–G). We further chose two nitrogen transporter genes (ammonium transporter*-AMT3;2* and nitrate transporter*-NRT1.2*) and six N-metabolism genes (*nitrate reductase-NR, glutamine dehydrogenase-GDH*, *glutamine synthetase-OsGS1; 1/OsGS1;2*) and *ferredoxin-dependent glutamate synthase1-GOGAT1/GOGAT2*) (Kurai et al., 2011). Quantitative real-time PCR analyses indicated that the expression of *OsAMT3;2* and *OsGS1;1* was upregulated and that *OsNRT1;2*, *NR*, and *OsGOGAT1* were downregulated in the *OsDOF11* mutant plants. Transcript level of *OsGDH*, *OsGS1;1* and *OsGOGAT1* were not obviously affected (Supplementary Figure 4). These results suggested that *OsDOF11* affects N signaling.

## *OsDOF11* Mediates Amino Metabolism in Flag Leaf

**FIGURE 6:**
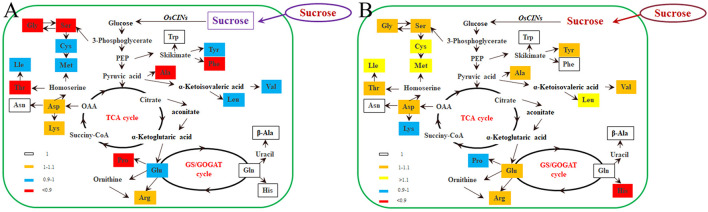
*OsDOF11* affects nitrogen metabolism by sugar distribution in flag leaves and seeds. **(A)** Free amino acid content in flag leaves; **(B)** free amino acid content in seeds.

In rice, flag leaves act as an important source to export and use photosynthates for grain filling (Ainsworth and Bush, 2011). Assessment of the concentration of N-containing metabolites (free amino acids) per gram of fresh weight revealed differences in the free amino acid content of flag leaves. The most significant change was reduction in the threonine (Thr), serine (Ser), glutamic acid (Glu), glycine (Gly), alanine (Ala), valine (Val), leucine (Leu), phenylalanine (Phe), and proline (Pro) concentrations in the flag leaves of *OsDOF11* mutant rice. Calculation of the amounts of these amino acids revealed that the total amino acids (TAAs), Essential Amino Acids (EAAs), and nonessential amino acids (NAAs) were all decreased (Figure 6A and Supplementary Table 2).

Leaf acts as the source, but the grain is a sink tissue. Previously, we reported that the *OsDOF11* mutant displayed a smaller grain size (Wu et al., 2018). However, the total starch and apparent amylose contents were not changed in flag leaves (Supplementary Table 5). Therefore, *OsDOF11* does not affect the starch percent in grains. We further measured the amino acid content in rice grains of *OsDOF11* mutants. Interestingly, the results demonstrated that aspartic acid (Asp), valine (Val), methionine (Met), isoleucine (Ile), leucine (Leu), and arginine (Arg) were obviously increased, as were TAAs, EAAs, and nonessential amino acids (NAAs) (Figure 6B and Supplementary Table 3). These results indicate that there are obvious differences between the C and N distributions in leaves and grains during rice development.

## Discussion

Previously, we studied *OsDOF11* genes and noted that *OsDOF11* mediated sucrose transport by binding the promoters of sucrose transporter genes. Here, we further studied the responses of N metabolism to sucrose, aiming to characterize physiological responses and metabolic pathways associated with the carbon distribution in *OsDOF11* mutant and *RNAi-9* rice plants. In conclusion, *OsDOF11* promotes sucrose transport to coordinate with C and N balance to maintain plant growth activity.

### OsDOF11 Involves Nitrogen Assimilation by Sucrose Transport

Reciprocal control between C and N uptake or assimilation coordinating the production of sugars and amino acids, according to the plant requirements, has been postulated (Reguera et al., 2013). We found that *OsDOF11* mutant plants took up less nitrogen than WT plants in Yoshida medium with multiple nitrogen contents at the seedling stage. *OsDOF11* mutant plants were grown in paddy fields with different concentrations of N by urea. Compared with the WT, the effective leaf blades of the *OsDOF11* mutants had lower N content, which was similar to the seedling stage. By transcriptome analysis, fundamental amino acid metabolism-related genes were affected, especially amino acid metabolism-related genes. Total amino acids, essential amino acids, and nonessential amino acids were all decreased in the leaf blade. Interestingly, total amino acids, essential amino acids, and nonessential amino acids were increased in *OsDOF11* seeds.

In the leaf blade, the soluble sugar contents in the leaf blade were not changed, but reduced in leaf sheath in the *OsDOF11* mutant plants (Wu et al., 2018) or in seeds. As we added 3% sucrose to Yoshida medium with multiple nitrogen contents, nitrogen uptake activity was increased. We reported that *OsDOF11* modulates sugar transport by regulating the expression of both *SUT* and *SWEET* genes in rice. The ratio of fresh weight to dry weight and nitrogen content in the *OsSWEET14* mutant at the seedling stage were reduced under multiple concentrations of nitrogen. Previously, ADP-glucose pyrophosphorylase (AGPase) was shown to control a rate-limiting step in the starch biosynthetic pathway. Both *OsAGPS2* and *OsAGPL2* knockout plants showed increased levels of free amino acids and soluble sugars in the endosperm, separately expressed in the leaf and endosperm (Lee et al., 2015). We have shown that reduced sucrose transport activity, mediated by mutation of the *OsDOF11* positive role in the inducement of sucrose transporter genes, led to lower content of sucrose in leaves and smaller size of the sink tissues (Wu et al., 2018). During the germination stage and flowering stage, *OsDOF11* is involved in N uptake. As the N concentration increases, the activity is enhanced. In addition, sucrose transport blockade of the loss of function of *OsDOF11* resulted in reduced efficiency of amino acid biosynthesis in leaves but enhanced N or amino acid content in seeds. All these results supported that sucrose levels promote nitrogen assimilation.

### Water Content Involves Susceptibility to Stress

As in previous reports, the sugar transporter genes *OsSWEET11*, *OsSWEET12*, *OsSWEET13*, *OsSWEET14*, and *OsSWEET15* are involved in responses to pathogen infection by inducing the secretion of sucrose into the apoplasm, where the pathogen grows (Wu et al., 2018, 2019). *OsDOF11* mediates susceptibility to infection by *Xanthomonas oryzae* pathovar *oryzae* and *Rhizoctonia solani* by *SWEET* genes (Wu et al., 2018; Kim et al., 2021). N and C are also two of the most important components for living organisms. N nutrition plays diverse roles in osmotic regulatory (Gao et al., 2016), soil drying (Zhong et al., 2018), drought (Gao et al., 2010), high temperatures (Liu et al., 2019). It is also reported that *OsGS2* performs important roles in the carbon-nitrogen metabolic balance in rice growth (Bao et al., 2015), which also contributes to improved drought tolerance (Singh and Ghosh, 2013). Both sugars and amino acids may affect the water content in rice plants, which might further mediate tissue metabolism activity and then lead to a stress response. Additionally, at the seedling stage and filling stage, the ratio of fresh weight to dry weight decreased in *OsDOF11* mutants, indicating that *OsDOF11* mediates water content by soluble sugars and amino acids, which further involves plant tissue metabolism activity. And Zhong et al. noted that coordinated regulation of the C and N metabolism facilitated the acclimation of rice photosynthesis to water deficit stress (Zhong et al., 2019). *OsDOF11* is expressed in the vasculature of roots, leaves, stems, and developing seeds. We found that the transcription levels of ammonium/nitrate transporter genes were affected in *OsDOF11-*related lines. Therefore, we will try to analyze whether there is crosstalk between ammonium/nitrate and sucrose transport, and whether N signaling or metabolism is involved in susceptibility to stress by *OsDOF11* in future work, which might provide new insight into the maintenance of carbon and nitrogen.

## Data Availability Statement

The datasets presented in this study can be found in online repositories. The names of the repository/repositories and accession number(s) can be found in the article/Supplementary Material.

## Author Contributions

YW, FX, GA, GC, and MJ designed the project. XH, YZ, LW, XD, and WH performed the experiments. WH, FX, GA, GC, MJ, XH, and YZ analyzed and interpreted the data. WH, FX, and GA wrote the manuscript with significant input from all authors. All authors contributed to the article and approved the submitted version.

## Conflict of Interest

The authors declare that the research was conducted in the absence of any commercial or financial relationships that could be construed as a potential conflict of interest.

## Publisher’s Note

All claims expressed in this article are solely those of the authors and do not necessarily represent those of their affiliated organizations, or those of the publisher, the editors and the reviewers. Any product that may be evaluated in this article, or claim that may be made by its manufacturer, is not guaranteed or endorsed by the publisher.

## Footnotes

1
http://ricephylogenomics-khu.org/ROAD/analysis/go_enrichment.shtml

## Supplementary Material

Supplementary Figure 1 Characterization of the *OsDOF11* mutants at the seedling stage under multiple N contents of Yoshida medium with 3% sucrose. **(A–H)** Phenotypes of the *osdof11-1* mutant, *OsDOF11-RNAi* plants, and WT under multiple N concentrations of Yoshida medium. **(A)** Picture of seedling plants at 7 days after germination; **(B)** shoot length; **(C)** root length; **(D)** root number; **(E)** ratio of shoot to root length; **(F)** fresh weight; **(G)** dry weight, **(H)** ratio of fresh weight to dry weight; **(I)** N concentration. 0 N: Yoshida medium without nitrogen, 0.1 N: Yoshida medium with 10% nitrogen, and normal medium: Yoshida medium with a normal content of nitrogen. Scale bar = 1 cm. Error bars represent SE of at least five samples. ^∗^*P* < 0.05; ^∗∗^*P* < 0.01.

Supplementary Figure 2 Characterization of the *OsSWEET14* Mutant. **(A–F)** Phenotypes of the *OsSWEET14* mutant and WT. **(C)** 110-day-old plants grown in the greenhouse; **(B–F)** panicle size and seed size. Scale bar, **(A)** 10 cm. **(B–C)** 1 cm Error bars represent SE of at least five samples. ^∗^*P* < 0.05; ^∗∗^*P* < 0.01.

Supplementary Figure 3 Top 20 of pathway enrichment.

Supplementary Figure 4 Expression levels of N assimilation genes relative to *OsUBQ5* evaluated at the flowering stage in the leaf blade. Transcript levels of *OsNRT1;2* **(A)**; *OsAMT3;2* **(B)**; *OsNR* **(C)**; *OsGDH* **(D)**; *OsGS1;1* **(E)**; *OsGS1;2* **(F)**; *OsGOGAT1* **(G)**; *OsGOGAT2* **(H)**. Each data point represents the mean (± SE) from at least four different plants. ^∗^*P* < 0.05; ^∗∗^*P* < 0.01.

Supplementary Figure 5 Sugar contents in *OsDOF11* mutant seeds. **(A)** Total starch content based on dry weight; **(B)** apparent amylose content based on dry weight. Each data point represents the mean (±SE) from at least four different samples.

Supplementary Table 1 Microarray analysis of amino acid metabolism-related genes in flag leaves.

Supplementary Table 2 Free amino acid content in flag leaf.

Supplementary Table 3 Free amino acid content in seed.

## References

[B1] Ainsworth E. A. Bush D. R. (2011). Carbohydrate export from the leaf: a highly regulated process and target to enhance photosynthesis and productivity. *Plant Physiol.* 155 64–69. 10.1104/pp.110.167684 20971857 PMC3075787

[B2] Antony G. Zhou J. Huang S. Li T. Liu B. White F. (2010). Rice xa13 recessive resistance to bacterial blight is defeated by induction of the disease susceptibility gene *Os-11N3*. *Plant Cell* 22 3864–3876. 10.1105/tpc.110.078964 21098734 PMC3015117

[B3] Ayre B. G. (2011). Membrane-transport systems for sucrose in relation to whole-plant carbon partitioning. *Mol. Plant* 4 377–394. 10.1093/mp/ssr014 21502663

[B4] Bai A. N. Lu X. D. Li D. Q. Liu J. X. Liu C. M. (2016). NF-YB1-regulated expression of sucrose transporters in aleurone facilitates sugar loading to rice endosperm. *Cell Res.* 26 384–388. 10.1038/cr.2015.116 26403192 PMC4783462

[B5] Bao A. Zhao Z. Q. Ding G. D. Shi L. Xu F. S. Cai H. M. (2015). The stable level of glutamine synthetase 2 plays an important role in rice growth and in carbon-nitrogen metabolic balance. *Int. J. Mol. Sci.* 16 12713–12736. 10.3390/ijms160612713 26053400 PMC4490469

[B6] Braun D. M. Wang L. Ruan Y. L. (2014). Understanding and manipulating sucrose phloem loading, unloading, metabolism, and signalling to enhance crop yield and food security. *J. Exp. Bot.* 65 1713–1735. 10.1093/jxb/ert416 24347463

[B7] Cao P. Jung K. H. Choi D. Hwang D. Zhu J. Ronald P. C. (2012). The rice oligonucleotide array database: an atlas of rice gene expression. *Rice* 5:17. 10.1186/1939-8433-5-17 24279809 PMC4883718

[B8] Coruzzi G. M. Zhou L. (2001). Carbon and nitrogen sensing and signaling in plants: emerging ‘matrix effects’. *Curr. Opin. Plant Biol.* 4 247–253. 10.1016/s1369-5266(00)00168-0 11312136

[B9] Eom J. S. Choi S. B. Ward J. M. Jeon J. S. (2012). The mechanism of phloem loading in rice (*Oryza sativa*). *Mol. Cells* 33 431–438. 10.1007/s10059-012-0071-9 22453778 PMC3887736

[B10] Fitzgerald M. A. McCouch S. R. Hall R. D. (2009). Not just a grain of rice: the quest for quality. *Trends Plant Sci.* 14 133–139. 10.1016/j.tplants.2008.12.004 19230745

[B11] Feng Y. X. Yu X. Z. Mo C. H. Lu C. J. (2019). Regulation network of sucrose metabolism in response to trivalent and hexavalent chromium in *Oryza sativa*. *J. Agr. Food Chem.* 67 9738–9748. 10.1021/acs.jafc.9b01720 31411877

[B12] Gao L. M. Liu M. Wang M. Shen Q. R. Guo S. W. (2016). Enhanced salt tolerance under nitrate nutrition is associated with apoplast Na + content in canola (*Brassica. napus* L.) and rice (*Oryza sativa* L.) plants. *Plant Cell Physiol.* 57 2323–2333. 10.1093/pcp/pcw141 27519313

[B13] Gao Y. X. Li Y. Yang X. X. Li H. J. Shen Q. R. Guo S. W. (2010). Ammonium nutrition increases water absorption in rice seedlings (*Oryza sativa* L.) under water stress. *Plant Soil* 331 193–201. 10.1007/s11104-009-0245-1

[B14] Jeon J. S. Lee S. Jung K. H. Jun S. H. Jeong D. H. Lee J. (2000). T-DNA insertional mutagenesis for functional genomics in rice. *Plant J.* 22 561–570. 10.1046/j.1365-313x.2000.00767.x 10886776

[B15] Jeong D. H. An S. Park S. Kang H. G. Park G. G. Kim S. R. (2006). Generation of a flanking sequence-tag database for activation-tagging lines in japonica rice. *Plant J.* 45 123–132. 10.1111/j.1365-313X.2005.02610.x 16367959

[B16] Julius B. T. Leach K. A. Tran T. M. Mertz R. A. Braun D. M. (2017). Sugar transporters in plants: new insights and discoveries. *Plant Cell Physiol.* 58 1442–1460. 10.1093/pcp/pcx090 28922744

[B17] Kim P. Xue C. Y. Song H. D. Gao Y. Feng L. Li Y. (2021). Tissue-specific activation of DOF11 promotes rice resistance to sheath blight disease and increases grain weight via activation of sweet14. *Plant Biotechnol. J.* 19 409–411. 10.1111/pbi.13489 33047500 PMC7955873

[B18] Kurai T. Wakayama M. Abiko T. Yanagisawa S. Aoki N. Ohsugi R. (2011). Introduction of the ZmDof1 gene into rice enhances carbon and nitrogen assimilation under low-nitrogen conditions. *Plant Biotechnol. J.* 9 826–837. 10.1111/j.1467-7652.2011.00592.x 21624033

[B19] Lee D. W. Lee S. K. Phee B. K. Jeon J. S. (2015). Proteomic analysis of the rice endosperm starch-deficient mutants *osagps2* and *osagpl2*. *J. Plant Biol.* 58 252–258. 10.1007/s12374-015-0160-3

[B20] Liu K. Deng J. Lu J. Wang X. Y. Lu B. L. Tian X. H. (2019). High nitrogen levels alleviate yield loss of super hybrid rice caused by high temperatures during the flowering stage. *Front. Plant Sci.* 10:357. 10.3389/fpls.2019.00357 30972091 PMC6443885

[B21] Mariotti F. Tomé D. Mirand P. P. (2008). Converting nitrogen into protein – beyond 6.25 and Jones’ Factors. *Crit. Rev. Food Sci. Nutr.* 48 177–184. 10.1080/10408390701279749 18274971

[B22] Nakamura Y. (2002). Towards a better understanding of the metabolic system for amylopectin biosynthesis in plants: rice endosperm as a model tissue. *Plant Cell Physiol.* 43 718–725. 10.1093/pcp/pcf091 12154134

[B23] Nunes-Nesi A. Fernie A. R. Stitt M. (2010). Metabolic and signaling aspects underpinning the regulation of plant carbon nitrogen interactions. *Mol. Plant* 3 973–996. 10.1093/mp/ssq049 20926550

[B24] Reguera M. Peleg Z. Abdel-Tawab Y. M. Tumimbang E. B. Delatorre C. A. Blumwald E. (2013). Stress-induced cytokinin synthesis increases drought tolerance through the coordinated regulation of carbon and nitrogen assimilation in rice. *Plant Physiol.* 163 1609–1622. 10.1104/pp.113.227702 24101772 PMC3850209

[B25] Sasaki T. Burr B. (2000). International rice genome sequencing project: the effort to completely sequence the rice genome. *Curr. Opin. Plant Biol.* 3 138–141. 10.1016/s1369-5266(99)00047-3 10712951

[B26] Singh K. K. Ghosh S. (2013). Regulation of glutamine synthetase isoforms in two differentially drought-tolerance rice (*Oryza sativa* L.) cultivars under water deficit conditions. *Plant Cell Rep.* 32 183–193. 10.1007/s00299-012-1353-6 23070303

[B27] Stitt M. Scheibe R. Feil R. (1989). Response of photosynthetic electron transport and carbon metabolism to a sudden decrease of irradiance in the saturating or the limiting range. *Biochim. Biophys. Acta Bioenerg.* 973 241–249. 10.1016/S0005-2728(89)80428-1

[B28] Sun X. Ling S. Lu Z. Ouyang Y. Yao J. (2014). *OsNF-YB1*, a rice endosperm-specific gene, is essential for cell proliferation in endosperm development. *Gene* 551 214–221. 10.1016/j.gene.2014.08.059 25178525

[B29] Tamura W. Kojima S. Toyokawa A. Watanabe H. TabuchiKobayashi M. Hayakawa T. (2011). Disruption of a novel NADH-glutamate synthase2 gene caused marked reduction in spikelet number of rice. *Front. Plant Sci.* 2:57. 10.3389/fpls.2011.00057 22645542 PMC3355815

[B30] Thimm O. Bläsing O. Gibon Y. Nagel A. Meyer S. Kruger P. (2004). MAPMAN: a user-driven tool to display genomics data sets onto diagrams of metabolic pathways and other biological processes. *Plant J.* 37 914–939. 10.1111/j.1365-313x.2004.02016.x 14996223

[B31] Tian Z. Qian Q. Liu Q. Yan M. Liu X. Yan C. (2009). Allelic diversities in rice starch biosynthesis lead to a diverse array of rice eating and cooking qualities. *Proc. Natl. Acad. Sci. U.S.A.* 106 21760–21765. 10.1073/pnas.0912396106 20018713 PMC2793318

[B32] Wang S. Liu S. Wang J. Yokosho K. Zhou B. Yu Y. (2020). Simultaneous changes in seed size, oil content, and protein content driven by selection of SWEET homologues during soybean domestication. *Natl. Sci. Rev.* 7 1776–1786. 10.1093/nsr/nwaa110 PMC8290959 34691511

[B33] Wu Y. Lee S. Yoo Y. Wei J. Lee S. Jeon J. (2018). Rice transcription factor OsDOF11 modulates sugar transport by enhancing expression of sucrose transporter (SUT) and SWEET genes. *Mol. Plant* 11 833–845. 29656028 10.1016/j.molp.2018.04.002

[B34] Wu Y. Peng W. Xiong F. (2019). Sucrose transport involves in disease response to *Xanthomonas oryzae* pathovar oryzae. *Plant Signal. Behav.* 3:1656949. 10.1080/15592324.2019.1656949 31578915 PMC6866690

[B35] Wu Y. Yang W. Wei J. Yoon H. An G. (2017). Transcription factor OsDOF18 controls ammonium uptake by inducing ammonium transporters in rice root. *Mol. Cells* 40 178–185. 10.14348/molcells.2017.2261 28292004 PMC5386955

[B36] Yan C. J. Tian Z. X. Fang Y. W. Yang Y. C. Li J. Zeng S. Y. (2011). Genetic analysis of starch paste viscosity parameters in glutinous rice (*Oryza sativa* L.). *Theor. Appl. Genet.* 122 63–76. 10.1007/s00122-010-1423-5 20737264

[B37] Yang J. Luo D. Yang B. Frommer W. B. Eom J. S. (2018). SWEET11 and 15 as key players in seed filling in rice. *New Phytol.* 218 604–615. 10.1111/nph.15004 29393510

[B38] Yoshida S. Forno D. A. Cock J. H. Gomez K. A. (1976). *Laboratory Manual for Physiological Studies of Rice*, 3rd Edn. Los Baños: International Rice Research Institute, 61–64.

[B39] Zhang J. S. Zhou Z. Y. Bai J. J. Tao X. Wang L. Zhang H. (2020). Disruption of *MIR396e* and *MIR396f* improves rice yield under nitrogen-deficient conditions. *Natl. Sci. Rev.* 7 102–112. 10.1093/nsr/nwz142 PMC8288854 34692021

[B40] Zhong C. Bai Z. G. Zhu L. F. Zhang J. H. Zhu C. Q. Huang J. L. (2019). Nitrogen-mediated alleviation of photosynthetic inhibition under moderate water stress in rice (*Oryza sativa* L.). *Environ. Exp. Bot.* 157 269–282. 10.1016/j.envexpbot.2018.10.021

[B41] Zhong C. Cao X. C. Bai Z. G. Zhang J. H. Zhu L. F. Huang J. L. (2018). Nitrogen metabolism correlates with the acclimation of photosynthesis to short-term water stress in rice (*Oryza sativa* L.). *Plant Physiol. Biochem.* 125 52–62. 10.1016/j.plaphy.2018.01.024 29413631

