# Supplementary material for: OsDOF11 Affects Nitrogen Metabolism by Sucrose Transport Signaling in Rice (Oryza sativa L.)
Source: Front Plant Sci. 2021 Sep 10;12:703034. doi: 10.3389/fpls.2021.703034 (PMC8461328; doi:10.3389/fpls.2021.703034)
Supplement: Supplementary Figure 1 — Characterization of the OsDOF11 mutants at the seedling stage under multiple N contents of Yoshida medium with 3% sucrose. (A–H) Phenotypes of the osdof11-1 mutant, OsDOF11-RNAi plants, and WT under multiple N concentrations of Yoshida medium. (A) Picture of seedling plants at 7 days after germination; (B) shoot length; (C) root length; (D) root number; (E) ratio of shoot to root length; (F) fresh weight; (G) dry weight, (H) ratio of fresh weight to dry weight; (I) N concentration. 0 N: Yoshida medium without nitrogen, 0.1 N: Yoshida medium with 10% nitrogen, and normal medium: Yoshida medium with a normal content of nitrogen. Scale bar = 1 cm. Error bars represent SE of at least five samples. ∗P < 0.05; ∗∗P < 0.01. [file Data_Sheet_1.PDF]

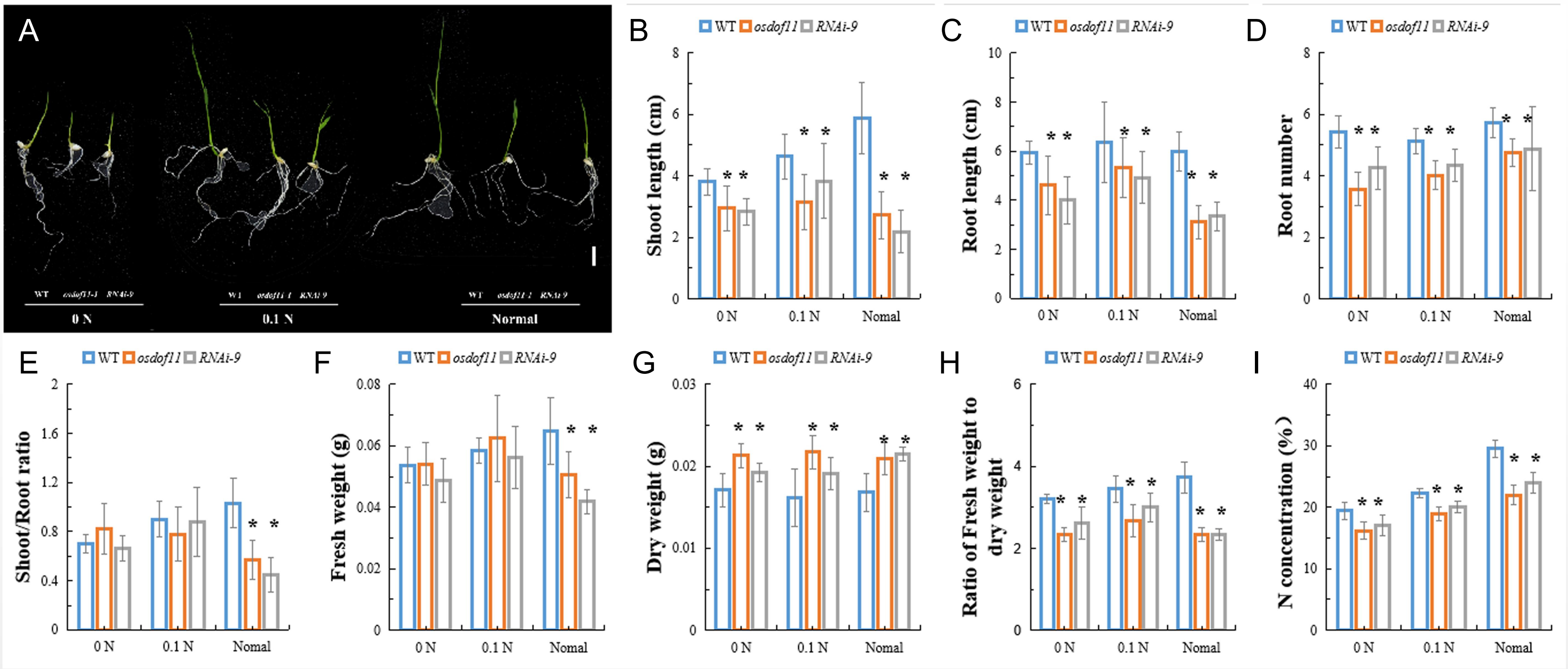

Supplemental Figure 1. Characterization of the *OsDOF11* mutants at the seedling stage under multiple N contents of Yoshida medium with 3% sucrose.

(A-H). Phenotypes of the *osdof11-1* mutant, *OsDOF11-RNAi* plants, and WT under multiple N concentrations of Yoshida medium. (A) Picture of seedling plants at 7 days after germination; (B) Shoot length; (C) Root length; (D) Root number; (E) Ratio of shoot to root length; (F) Fresh weight; (G) Dry weight, (H) Ratio of fresh weight to dry weight; (I) N concentration. 0 N: Yoshida medium without nitrogen, 0.1 N: Yoshida medium with 10% nitrogen, and normal medium: Yoshida medium with a normal content of nitrogen. Scale bar = 1 cm. Error bars represent SE of at least five samples. *P < 0.05.

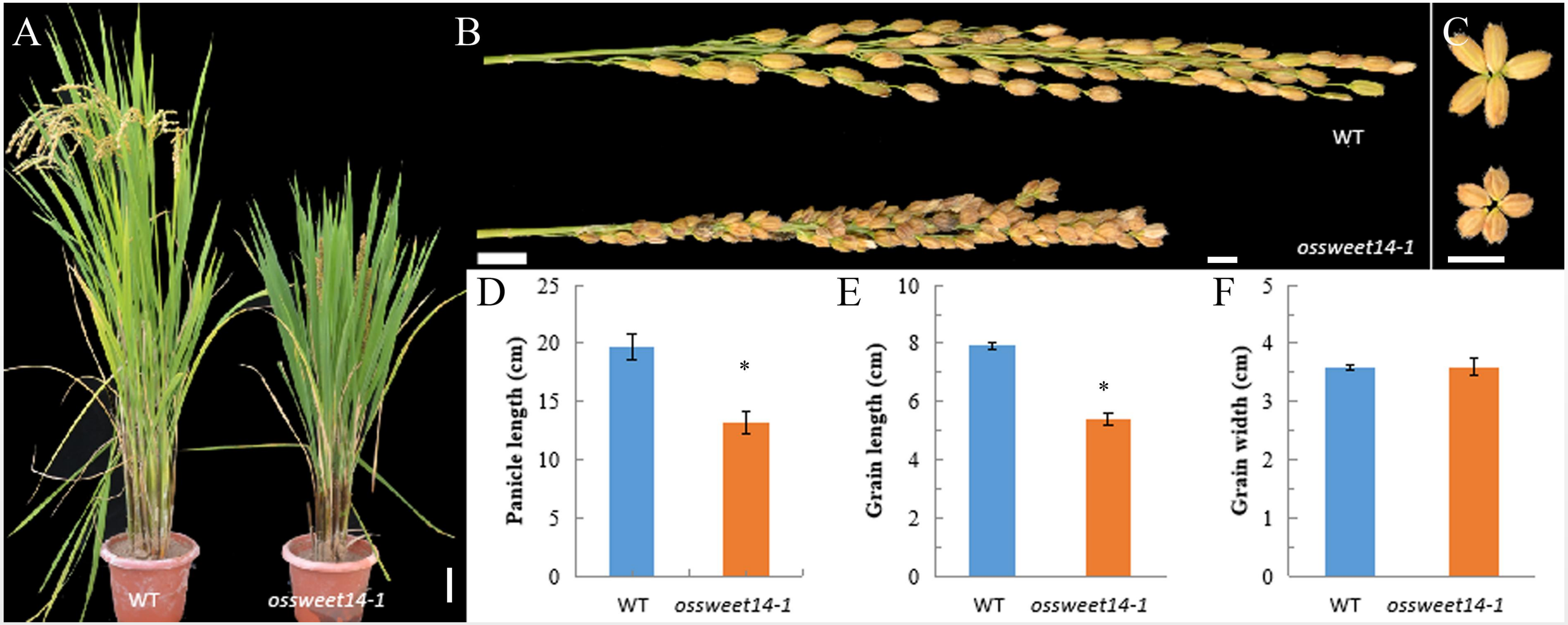

Supplemental Figure 2. Characterization of the *OsSWEET14* Mutant.
(A–F) Phenotypes of the *OsSWEET14* mutant and WT. (C) 110-day-old plants grown in the greenhouse; (B-F) Panicle size and seed size.
Scale bar, A: 10 cm. B-C: 1 cm Error bars represent SE of at least five samples. *P < 0.05.
Supplemental Figure 3. Top 20 of pathway enrichment.

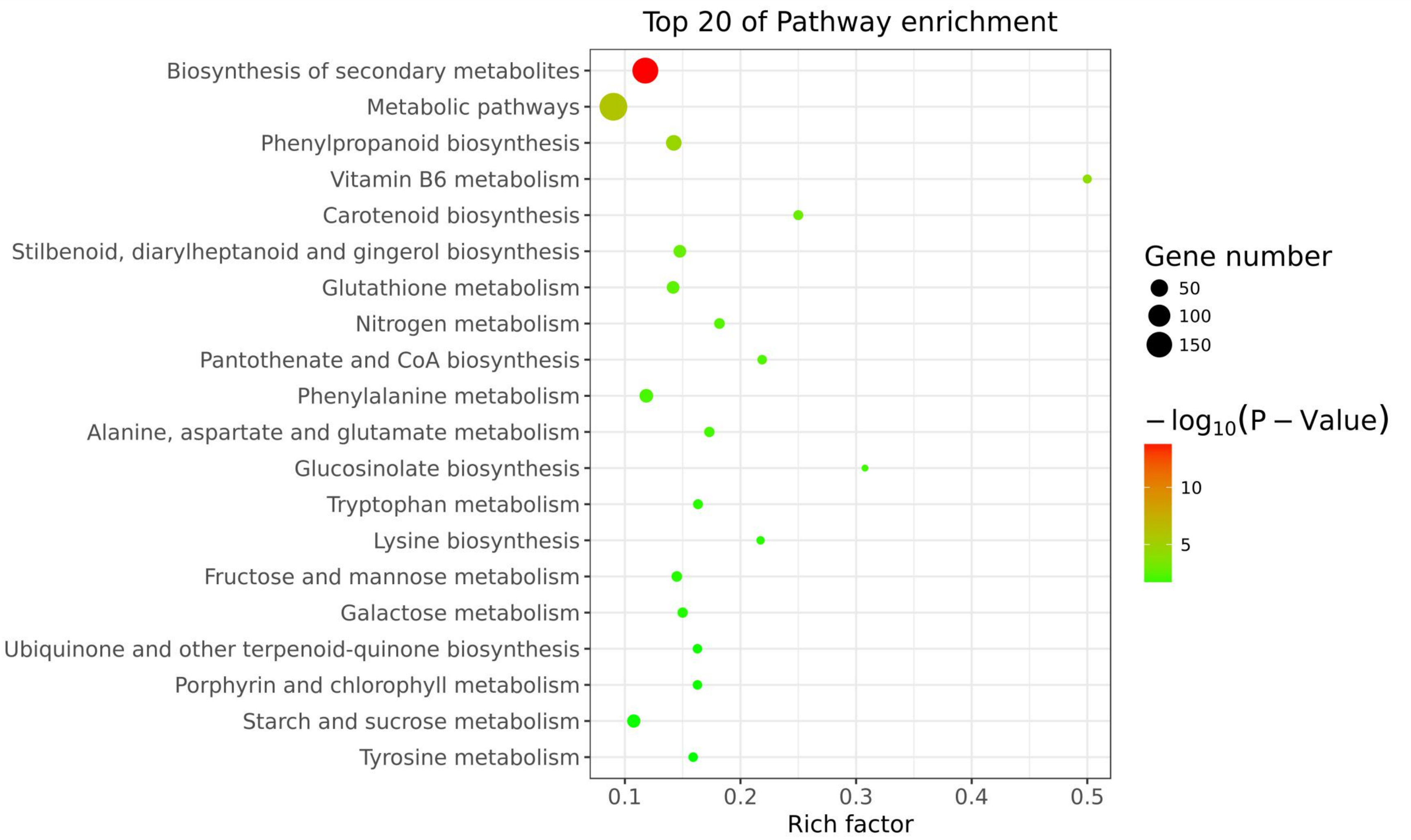

Supplemental Figure 3. Top 20 of pathway enrichment.

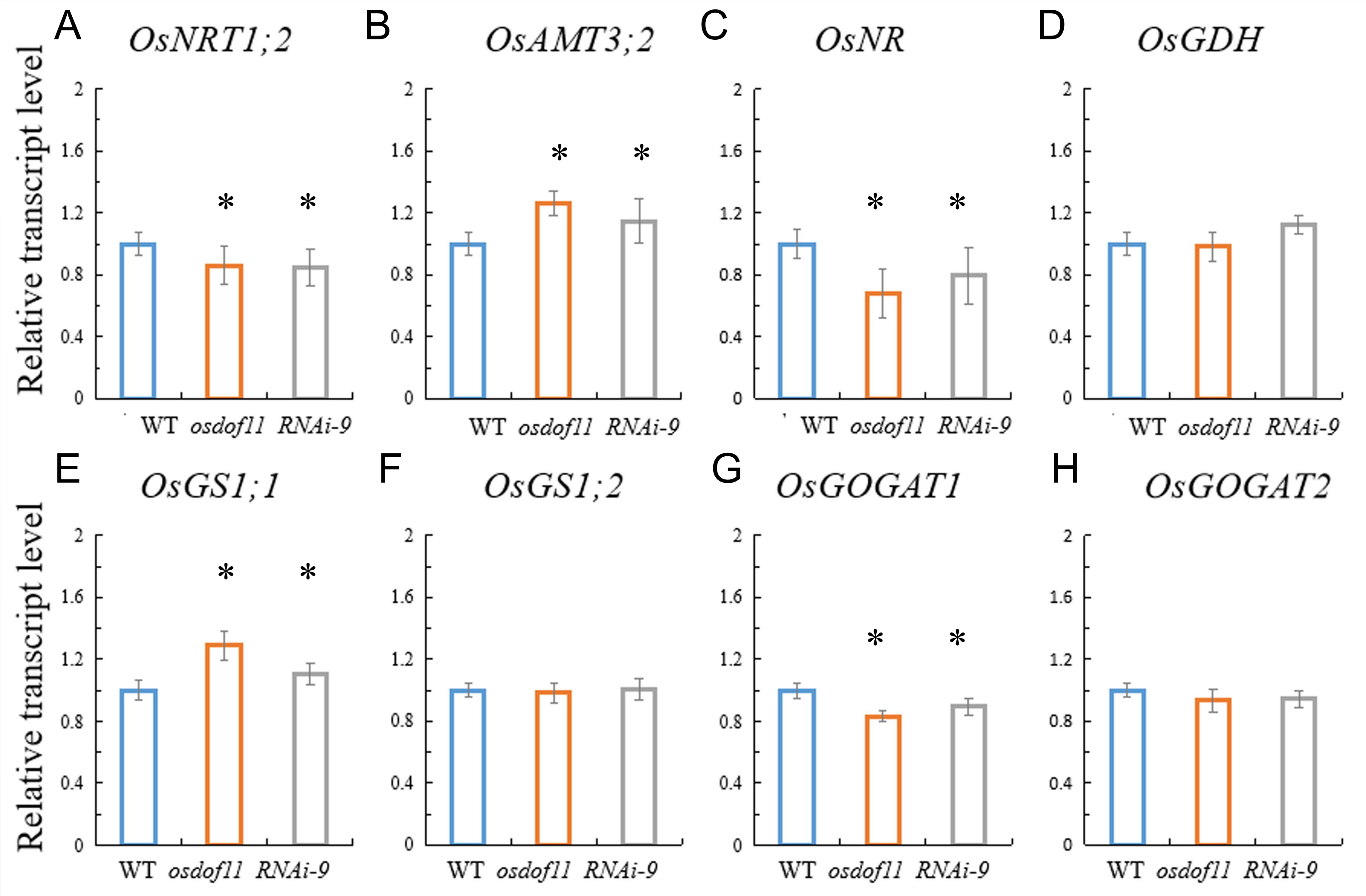

Supplemental Figure 4. Expression levels of N assimilation genes relative to *OsUBQ5* evaluated at the flowering stage in the leaf blade.

Transcript levels of *OsNRT1;2* (A); *OsAMT3;2* (B); O*sNR* (C); *OsGDH* (D); *OsGS1;1* (E); *OsGS1;2* (F); *OsGOGAT1* (G); *OsGOGAT2* (H). Each data point represents the mean (±SE) from at least four different plants. *, P <0.05.

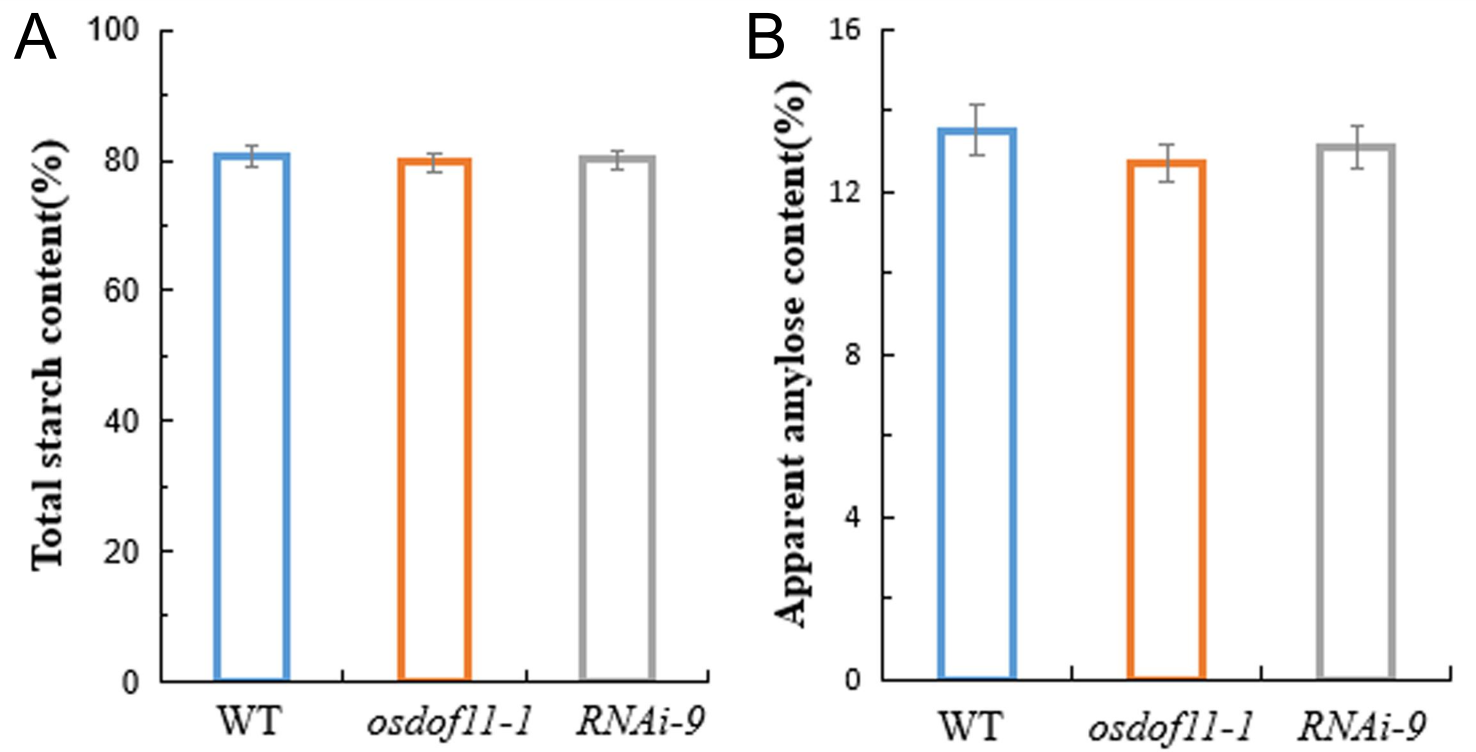

Supplemental Figure 5. Sugar contents in *OsDOF11* mutant seeds.
(A) Total starch content based on dry weight; (B) apparent amylose content based on dry weight. Each data point represents the mean (±SE) from at least four different samples..
