# Supplementary material for: OsDOF11 Affects Nitrogen Metabolism by Sucrose Transport Signaling in Rice (Oryza sativa L.)
Source: Front Plant Sci. 2021 Sep 10;12:703034. doi: 10.3389/fpls.2021.703034 (PMC8461328; doi:10.3389/fpls.2021.703034)
Supplement: Supplementary Table 1 — Microarray analysis of amino acid metabolism-related genes in flag leaves. [file Data_Sheet_2.docx]

Supplemental Table 1. RNA-sequence analysis of amino acid metabolism related genes in flag leaf.

| PathwayID | PathwayTerm | QueryID | Description | Enrichment |
| --- | --- | --- | --- | --- |
| PATH:00350 | Tyrosine metabolism | LOC_Os02g07160 | Similar to 4-hydroxyphenylpyruvate dioxygenase | -3.666039375 |
| PATH:00350 | Tyrosine metabolism | LOC_Os10g26110 | Similar to Pyridoxal-dependent decarboxylase conserved domain containing protein | -3.666039375 |
| PATH:00350 | Tyrosine metabolism | LOC_Os06g23684 | Similar to Nicotianamine aminotransferase | -3.666039375 |
| PATH:00350 | Tyrosine metabolism | LOC_Os06g01360 | Similar to Homogentisate 1,2-dioxygenase | -3.666039375 |
| PATH:00350 | Tyrosine metabolism | LOC_Os02g20360 | Similar to Nicotianamine aminotransferase A | -3.666039375 |
| PATH:00480 | Glutathione metabolism | LOC_Os10g22070 | Thioredoxin fold domain containing protein | -2.28622298 |
| PATH:00480 | Glutathione metabolism | LOC_Os03g17460 | Similar to In2-1 protein | -2.28622298 |
| PATH:00480 | Glutathione metabolism | LOC_Os01g49720 | Glutathione S-transferase, C-terminal-like domain containing protein | -2.28622298 |
| PATH:00480 | Glutathione metabolism | LOC_Os03g57200 | Similar to Glutathione-S-transferase. | -2.28622298 |
| PATH:00480 | Glutathione metabolism | LOC_Os01g49710 | Similar to Glutathione S-transferase GSTU6 | -2.28622298 |
| PATH:00480 | Glutathione metabolism | LOC_Os01g72130 | Similar to Glutathione S-transferase GST 28 | -2.28622298 |
| PATH:00480 | Glutathione metabolism | LOC_Os10g38610 | Similar to glutathione transferase35. | -2.28622298 |
| PATH:00480 | Glutathione metabolism | LOC_Os02g44500 | Similar to Phospholipid hydroperoxide glutathione peroxidase | -2.28622298 |
| PATH:00280 | Valine, leucine and isoleucine degradation | LOC_Os03g12890 | Similar to Branched-chain-amino-acid aminotransferase 3 | -3.22611465 |
| PATH:00280 | Valine, leucine and isoleucine degradation | LOC_Os09g26880 | Similar to Aldehyde dehydrogenase family 7 member A1 | -3.22611465 |
| PATH:00280 | Valine, leucine and isoleucine degradation | LOC_Os01g21160 | Catalytic domain of components of various dehydrogenase complexes containing protein | -3.22611465 |
| PATH:00280 | Valine, leucine and isoleucine degradation | LOC_Os01g02020 | Similar to Acetyl-CoA C-acetyltransferase. | -3.22611465 |
| PATH:00280 | Valine, leucine and isoleucine degradation | LOC_Os05g15530 | Aminotransferase | -3.22611465 |
| PATH:00250 | Alanine, aspartate and glutamate metabolism | LOC_Os03g12290 | Glutamine synthetase root isozyme | -3.102033317 |
| PATH:00250 | Alanine, aspartate and glutamate metabolism | LOC_Os01g65260 | Similar to Amidophosphoribosyltransferase | -3.102033317 |
| PATH:00250 | Alanine, aspartate and glutamate metabolism | LOC_Os05g39770 | Similar to Alanine:glyoxylate aminotransferase-like protein | -3.102033317 |
| PATH:00250 | Alanine, aspartate and glutamate metabolism | LOC_Os01g48960 | Non-protein coding transcript | -3.102033317 |
| PATH:00250 | Alanine, aspartate and glutamate metabolism | LOC_Os02g04170 | Fumarate reductase/succinate dehydrogenase flavoprotein | -3.102033317 |
| PATH:00260 | Glycine, serine and threonine metabolism | LOC_Os03g06200 | Phosphoserine aminotransferase, chloroplast precursor | -2.481626654 |
| PATH:00260 | Glycine, serine and threonine metabolism | LOC_Os11g04300 | Similar to Phosphoglycerate mutase family protein | -2.481626654 |
| PATH:00260 | Glycine, serine and threonine metabolism | LOC_Os09g26880 | Similar to Aldehyde dehydrogenase family 7 member A1 | -2.481626654 |
| PATH:00260 | Glycine, serine and threonine metabolism | LOC_Os05g39770 | Similar to Alanine:glyoxylate aminotransferase-like protein | -2.481626654 |
| PATH:00260 | Glycine, serine and threonine metabolism | LOC_Os04g43650 | Pyridoxal phosphate-dependent transferase | -2.481626654 |
| PATH:00380 | Tryptophan metabolism | LOC_Os09g26880 | Similar to Aldehyde dehydrogenase family 7 member A1 | -2.633562979 |
| PATH:00380 | Tryptophan metabolism | LOC_Os01g02020 | Similar to Acetyl-CoA C-acetyltransferase | -2.633562979 |
| PATH:00380 | Tryptophan metabolism | LOC_Os01g12490 | Flavin monooxygenase-like enzyme | -2.633562979 |
| PATH:00380 | Tryptophan metabolism | LOC_Os10g26110 | Similar to Pyridoxal-dependent decarboxylase conserved domain containing protein | -2.633562979 |
| PATH:00310 | Lysine degradation | LOC_Os09g26880 | Similar to Aldehyde dehydrogenase family 7 member A1 | -2.615768635 |
| PATH:00310 | Lysine degradation | LOC_Os01g02020 | Similar to Acetyl-CoA C-acetyltransferase | -2.615768635 |
| PATH:00310 | Lysine degradation | LOC_Os02g54254 | Similar to Lysine ketoglutarate reductase/saccharopine dehydrogenase | -2.615768635 |
| PATH:00400 | Phenylalanine, tyrosine and tryptophan biosynthesis | LOC_Os06g23684 | Similar to Nicotianamine aminotransferase | -2.08136429 |
| PATH:00400 | Phenylalanine, tyrosine and tryptophan biosynthesis | LOC_Os02g20360 | Similar to Nicotianamine aminotransferase A | -2.08136429 |
| PATH:00400 | Phenylalanine, tyrosine and tryptophan biosynthesis | LOC_Os10g37980 | Prephenate dehydratase domain containing protein | -2.08136429 |
| PATH:00400 | Phenylalanine, tyrosine and tryptophan biosynthesis | LOC_Os07g49390 | Prephenate dehydratase domain containing protein | -2.08136429 |
| PATH:00300 | Lysine biosynthesis | LOC_Os09g26880 | Similar to Aldehyde dehydrogenase family 7 member A1 | -2.805317087 |
| PATH:00300 | Lysine biosynthesis | LOC_Os02g54254 | Similar to Lysine ketoglutarate reductase/saccharopine dehydrogenase | -2.805317087 |
| PATH:00330 | Arginine and proline metabolism | LOC_Os09g26880 | Similar to Aldehyde dehydrogenase family 7 member A1 | -1.743845757 |
| PATH:00330 | Arginine and proline metabolism | LOC_Os03g12290 | Glutamine synthetase root isozyme | -1.743845757 |
| PATH:00330 | Arginine and proline metabolism | LOC_Os04g39210 | Agmatine deiminase domain containing protein | -1.743845757 |
| PATH:00330 | Arginine and proline metabolism | LOC_Os03g44150 | Similar to Ornithine-oxo-acid transaminase | -1.743845757 |
| PATH:00270 | Cysteine and methionine metabolism | LOC_Os10g37340 | Cys/Met metabolism | -1.613057325 |
| PATH:00270 | Cysteine and methionine metabolism | LOC_Os01g52260 | Similar to satase isoform I | -1.613057325 |
| PATH:00270 | Cysteine and methionine metabolism | LOC_Os03g10050 | Similar to satase isoform II | -1.613057325 |
| PATH:00270 | Cysteine and methionine metabolism | LOC_Os06g23684 | Similar to Nicotianamine aminotransferase | -1.613057325 |
| PATH:00270 | Cysteine and methionine metabolism | LOC_Os02g20360 | Similar to Nicotianamine aminotransferase A | -1.613057325 |
| PATH:00340 | Histidine metabolism | LOC_Os09g26880 | Similar to Aldehyde dehydrogenase family 7 member A1 | -2.224906655 |
| PATH:00340 | Histidine metabolism | LOC_Os10g26110 | Similar to Pyridoxal-dependent decarboxylase conserved domain containing protein | -2.224906655 |
| PATH:00410 | beta-Alanine metabolism | LOC_Os09g26880 | Similar to Aldehyde dehydrogenase family 7 member A1 | -1.697955079 |
| PATH:00410 | beta-Alanine metabolism | LOC_Os04g57550 | Similar to Suppressor of presenilin 5 (P110b homolog) | -1.697955079 |
| PATH:00290 | Valine, leucine and isoleucine biosynthesis | LOC_Os03g12890 | Similar to Branched-chain-amino-acid aminotransferase 3 | -1.500518442 |
| PATH:00290 | Valine, leucine and isoleucine biosynthesis | LOC_Os05g15530 | Aminotransferase, class IV family protein | -1.500518442 |
| PATH:00360 | Phenylalanine metabolism | LOC_Os02g46970 | Similar to 4-coumarate--CoA ligase 1 | -0.997767417 |
| PATH:00360 | Phenylalanine metabolism | LOC_Os02g07160 | Similar to 4-hydroxyphenylpyruvate dioxygenase | -0.997767417 |
| PATH:00360 | Phenylalanine metabolism | LOC_Os04g43800 | Similar to Phenylalanine ammonia-lyase | -0.997767417 |
| PATH:00360 | Phenylalanine metabolism | LOC_Os10g26110 | Similar to Pyridoxal-dependent decarboxylase conserved domain containing protein | -0.997767417 |
| PATH:00360 | Phenylalanine metabolism | LOC_Os06g23684 | Similar to Nicotianamine aminotransferase | -0.997767417 |
| PATH:00360 | Phenylalanine metabolism | LOC_Os02g20360 | Similar to Nicotianamine aminotransferase A | -0.997767417 |
| PATH:00360 | Phenylalanine metabolism | LOC_Os07g48010 | Peroxidase. | 2.287836114 |
| PATH:00360 | Phenylalanine metabolism | LOC_Os07g48020 | Similar to Peroxidase. | 2.287836114 |
| PATH:00360 | Phenylalanine metabolism | LOC_Os02g41680 | Similar to Phenylalanine ammonia-lyase | 2.287836114 |
| PATH:00360 | Phenylalanine metabolism | LOC_Os02g41650 | Phenylalanine ammonia-lyase | 2.287836114 |
| PATH:00360 | Phenylalanine metabolism | LOC_Os01g19020 | Haem peroxidase | 2.287836114 |
| PATH:00360 | Phenylalanine metabolism | LOC_Os03g55410 | Similar to Peroxidase 51 precursor | 2.287836114 |
| PATH:00360 | Phenylalanine metabolism | LOC_Os07g49360 | Similar to Peroxidase | 2.287836114 |
| PATH:00360 | Phenylalanine metabolism | LOC_Os05g41440 | Similar to Cytochrome P450 98A1 | 2.287836114 |
| PATH:00360 | Phenylalanine metabolism | LOC_Os08g38900 | Similar to Caffeoyl-CoA O-methyltransferase 2 | 2.287836114 |
| PATH:00360 | Phenylalanine metabolism | LOC_Os01g73200 | Similar to Peroxidase BP 1 precursor | 2.287836114 |
| PATH:00360 | Phenylalanine metabolism | LOC_Os07g48050 | Similar to Peroxidase precursor | 2.287836114 |
| PATH:00360 | Phenylalanine metabolism | LOC_Os02g41630 | Similar to Phenylalanine ammonia-lyase | 2.287836114 |
| PATH:00360 | Phenylalanine metabolism | LOC_Os05g04380 | Haem peroxidase, plant/fungal/bacterial family protein | 2.287836114 |
| PATH:00360 | Phenylalanine metabolism | LOC_Os01g73170 | Similar to Peroxidase 12 precursor | 2.287836114 |
| PATH:00360 | Phenylalanine metabolism | LOC_Os02g08100 | Similar to 4-coumarate--CoA ligase 1 | 2.287836114 |
| PATH:00360 | Phenylalanine metabolism | LOC_Os01g28030 | Plant peroxidase domain containing protein | 2.287836114 |
| PATH:00360 | Phenylalanine metabolism | LOC_Os01g22370 | Similar to Peroxidase | 2.287836114 |
| PATH:00480 | Glutathione metabolism | LOC_Os03g04260 | Similar to Glutathione S-transferase GST 10 | 1.850190762 |
| PATH:00480 | Glutathione metabolism | LOC_Os10g38360 | Similar to Tau class GST protein 3 | 1.850190762 |
| PATH:00480 | Glutathione metabolism | LOC_Os10g22310 | Similar to Glutathione S-transferase GSTU35 | 1.850190762 |
| PATH:00480 | Glutathione metabolism | LOC_Os12g16200 | Similar to Glutathione synthase. | 1.850190762 |
| PATH:00480 | Glutathione metabolism | LOC_Os04g35520 | Similar to Thylakoid-bound ascorbate peroxidase | 1.850190762 |
| PATH:00480 | Glutathione metabolism | LOC_Os09g29200 | Similar to Glutathione S-transferase GST 23 | 1.850190762 |
| PATH:00480 | Glutathione metabolism | LOC_Os04g40874 | Glucose-6-phosphate dehydrogenase | 1.850190762 |
| PATH:00480 | Glutathione metabolism | LOC_Os10g38780 | Glutathione S-transferase GST 30 | 1.850190762 |
| PATH:00480 | Glutathione metabolism | LOC_Os01g72170 | Conserved hypothetical protein. | 1.850190762 |
| PATH:00300 | Lysine biosynthesis | LOC_Os08g25390 | Similar to aspartate kinase-homoserine dehydrogenase | 3.405423577 |
| PATH:00300 | Lysine biosynthesis | LOC_Os03g18810 | Similar to Plastid aminotransferase | 3.405423577 |
| PATH:00300 | Lysine biosynthesis | LOC_Os03g63330 | Similar to Aspartokinase. | 3.405423577 |
| PATH:00290 | Valine, leucine and isoleucine biosynthesis | LOC_Os02g17330 | Aminotransferase, class IV family protein. | 2.428674179 |
| PATH:00290 | Valine, leucine and isoleucine biosynthesis | LOC_Os11g14950 | Hypothetical conserved gene. | 2.428674179 |
| PATH:00290 | Valine, leucine and isoleucine biosynthesis | LOC_Os08g44530 | Similar to Dihydroxy-acid dehydratase | 2.428674179 |
| PATH:00290 | Valine, leucine and isoleucine biosynthesis | LOC_Os01g46380 | Similar to Ketol-acid reductoisomerase | 2.428674179 |
| PATH:00380 | Tryptophan metabolism | LOC_Os04g08828 | Similar to Cytochrome P450 79A1 | 2.131285504 |
| PATH:00380 | Tryptophan metabolism | LOC_Os12g16720 | Similar to Cytochrome P450 71A1 | 2.131285504 |
| PATH:00380 | Tryptophan metabolism | LOC_Os02g02400 | Catalase isozyme A | 2.131285504 |
| PATH:00380 | Tryptophan metabolism | LOC_Os04g08824 | Similar to Cytochrome P450 79A1 | 2.131285504 |
| PATH:00250 | Alanine, aspartate and glutamate metabolism | LOC_Os12g13320 | Similar to Argininosuccinate synthase | 2.008326725 |
| PATH:00250 | Alanine, aspartate and glutamate metabolism | LOC_Os03g19280 | Fumarate lyase domain containing protein | 2.008326725 |
| PATH:00250 | Alanine, aspartate and glutamate metabolism | LOC_Os08g36320 | Similar to Glutamate decarboxylase | 2.008326725 |
| PATH:00250 | Alanine, aspartate and glutamate metabolism | LOC_Os03g18130 | Similar to Asparagine synthetase | 2.008326725 |
| PATH:00330 | Arginine and proline metabolism | LOC_Os12g13320 | Similar to Argininosuccinate synthase | 1.764070772 |
| PATH:00330 | Arginine and proline metabolism | LOC_Os03g19280 | Fumarate lyase domain containing protein | 1.764070772 |
| PATH:00330 | Arginine and proline metabolism | LOC_Os03g31690 | Similar to N-acetyl-glutamate synthase | 1.764070772 |
| PATH:00330 | Arginine and proline metabolism | LOC_Os03g17120 | Similar to predicted protein | 1.764070772 |
| PATH:00330 | Arginine and proline metabolism | LOC_Os02g47590 | Similar to ornithine carbamoyltransferase | 1.764070772 |
| PATH:00260 | Glycine, serine and threonine metabolism | LOC_Os08g25390 | Similar to aspartate kinase-homoserine dehydrogenase. | 1.60666138 |
| PATH:00260 | Glycine, serine and threonine metabolism | LOC_Os05g47640 | Similar to Threonine synthase | 1.60666138 |
| PATH:00260 | Glycine, serine and threonine metabolism | LOC_Os02g58510 | Similar to 3-hydroxy-3-methylglutaryl-coenzyme A reductase 2 | 1.60666138 |
| PATH:00260 | Glycine, serine and threonine metabolism | LOC_Os03g63330 | Similar to Aspartokinase | 1.60666138 |
| PATH:00340 | Histidine metabolism | LOC_Os01g16940 | Similar to Histidine biosynthesis bifunctional protein hisIE | 1.800568788 |
| PATH:00340 | Histidine metabolism | LOC_Os03g15120 | Similar to Imidazole glycerol phosphate synthase hisHF | 1.800568788 |
| PATH:00270 | Cysteine and methionine metabolism | LOC_Os08g25390 | Similar to aspartate kinase-homoserine dehydrogenase | 1.305412371 |
| PATH:00270 | Cysteine and methionine metabolism | LOC_Os01g18860 | Similar to S-adenosylmethionine synthetase 1 | 1.305412371 |
| PATH:00270 | Cysteine and methionine metabolism | LOC_Os11g26850 | Similar to Adenosylhomocysteinase. | 1.305412371 |
| PATH:00270 | Cysteine and methionine metabolism | LOC_Os05g05680 | Similar to 1-aminocyclopropane-1-carboxylic acid oxidase | 1.305412371 |
| PATH:00270 | Cysteine and methionine metabolism | LOC_Os03g63330 | Similar to Aspartokinase | 1.305412371 |
| PATH:00350 | Tyrosine metabolism | LOC_Os07g42924 | Alcohol dehydrogenase superfamily | 1.186738519 |
| PATH:00350 | Tyrosine metabolism | LOC_Os11g10520 | Similar to Alcohol dehydrogenase | 1.186738519 |
| PATH:00400 | Phenylalanine, tyrosine and tryptophan biosynthesis | LOC_Os07g42960 | Similar to M-160-u1_1 | 0.84220153 |
| PATH:00400 | Phenylalanine, tyrosine and tryptophan biosynthesis | LOC_Os03g27230 | Similar to Phospho-2-dehydro-3-deoxyheptonate aldolase 1 | 0.84220153 |
| PATH:00410 | beta-Alanine metabolism | LOC_Os08g36320 | Similar to Glutamate decarboxylase | 0.687059143 |

Supplemental Table 2. Free amino acid content in flag leaf.


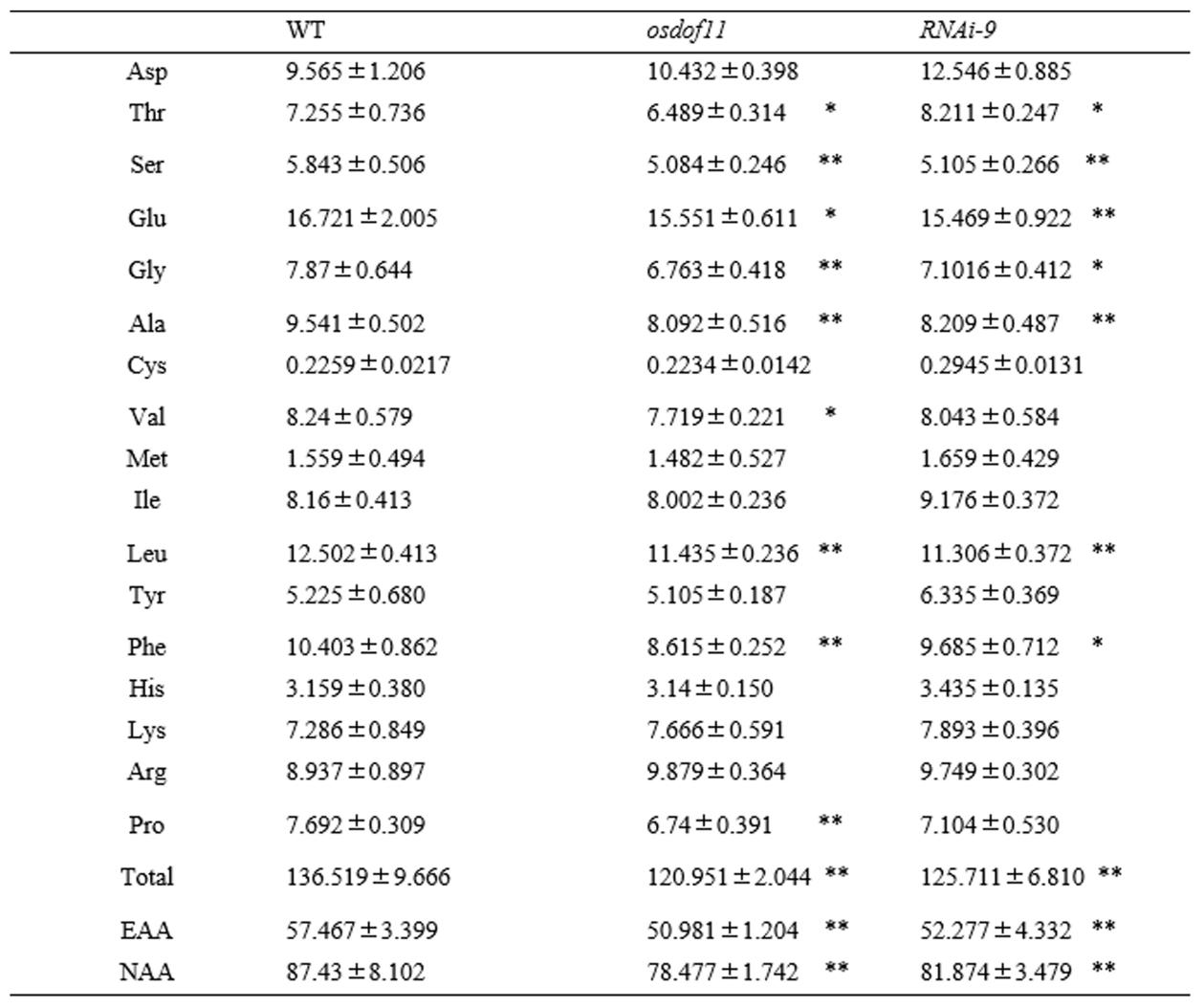


Supplemental Table 3. Free amino acid content in seed


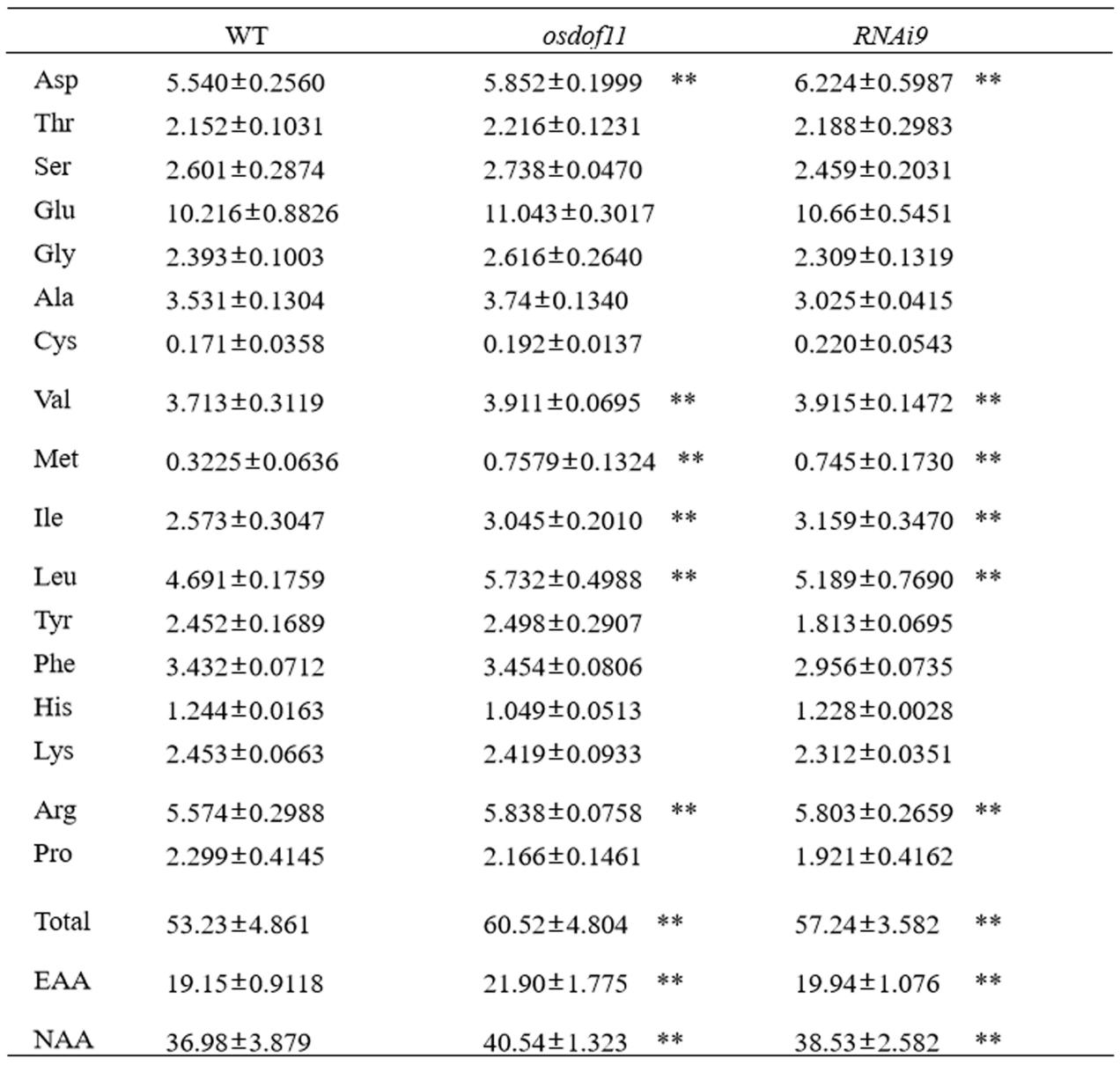


Supplementary Table 4 List of PCR primers used in this study

| Gene |  | Sequence (5'-3') | Gene-ID |
| --- | --- | --- | --- |
| Ammonium transporter (AMT3;2) | Forward | CCATGATGATCCTGCACAAGA | LOC_Os03g62200 |
|  | Reverse | CGGCGTGGGTGTGGAA |  |
| Nitrate transporter (NRT1.2) | Forward | GGAGAGGTTCTACTGGGTGATGTG | LOC_Os06g38294 |
|  | Reverse | GATGGCCCAGAACAAGAAGAAG |  |
| Nitrate Reductase (NR) 2 | Forward | CGAGAAGCTCATCTGGAATCTCA | LOC_Os08g36480 |
|  | Reverse | AGCCCGATCTCACCCTTGT |  |
| Glutamine dehydrogenase (GDH) | Forward | GTTCTCATCCCATGCGCTTTAG | LOC_Os04g45970 |
|  | Reverse | GCCTTCACATCAGGTGCATTT |  |
| glutamine synthetase (OsGS1;1) | Forward | CGGAGTCGTCGTCTCATTTG | [LOC_Os02g50240](http://rice.plantbiology.msu.edu/cgi-bin/ORF_infopage.cgi?orf=LOC_Os02g50240) |
|  | Reverse | TCTCAATGGCGGACTTGATG |  |
| glutamine synthetase (OsGS1;2) | Forward | CCCCTTCACCGACAAGATCA | LOC_Os04g56400 |
|  | Reverse | AGGTCAATTCCAGTTCCTCCAA |  |
| ferredoxin-dependent glutamate synthase1 (OsNADH-GOGAT1) | Forward | GTGCAGCCTGTTGCAGCATAAA | [LOC_Os07g46460](http://rice.plantbiology.msu.edu/cgi-bin/ORF_infopage.cgi?orf=LOC_Os07g46460) |
|  | Reverse | CGGCATTTCACCATGCAAATC |  |
| ferredoxin-dependent glutamate synthase1 (OsNADH-GOGAT2) | Forward | GAAGGATGATGAAGGTGAAACC | [LOC_Os05g48200](http://rice.plantbiology.msu.edu/cgi-bin/ORF_infopage.cgi?orf=LOC_Os05g48200) |
|  | Reverse | ATGGCCCTACTATCTTCGCAT |  |
| OsUBQ5 | Forward | TGAAGACCCTGACTGGGAAG | LOC_Os01g22490 |
|  | Reverse | CACGGTTCAACAACATCCAG |  |
| LOC_Os04g43800 |  | GCTCTTCCGCACACACAACTC |  |
|  |  | TTGCTCGAGCGGTTTGC |  |
| LOC_Os02g34600 |  | TCGGGTCGGGCAACTT |  |
|  |  | CATCCAGCAGCGTGTTCT |  |
| LOC_Os02g41680 |  | CGGTGTTGTTTTTATCTGGTGAAT |  |
|  |  | GCCGCTATGCAACGAAGAA |  |
| LOC_Os02g41650 |  | GCATCAGCTTCCAACTCG |  |
|  |  | GGTTTCGCACTCCATTACAGA |  |
| LOC_Os09g29200 |  | AGCAGAGAAAGCTGGTGCAC |  |
|  |  | CATCAGGGGCAGCGCCTC |  |
| LOC_Os02g41630 |  | CGCCATGGCCTCCTACTG |  |
|  |  | GCTCTGGACATGGTTGGTGAT |  |
